# Hyperleptinemia directly affects testicular maturation at different sexual stages in mice, and suppressor of cytokine signaling 3 is involved in this process

**DOI:** 10.1186/1477-7827-12-15

**Published:** 2014-02-06

**Authors:** Miao Yuan, Guizhen Huang, Jun Li, Jie Zhang, Fei Li, Kai Li, Bo Gao, Li Zeng, Wei Shan, Ping Lin, Lugang Huang

**Affiliations:** 1 Department of Pediatric Surgery, West China Hospital, Sichuan University, Chengdu, P.R. China; 2 Division of Geriatrics, State Key Laboratory of Biotherapy, West China Hospital, Sichuan University, Chengdu, P.R. China

**Keywords:** MSG, Leptin, Testis, Testosterone, SOCS3, pSTAT3

## Abstract

**Background:**

Leptin plays an important role in reproductive function, and the mechanism of this phenomenon primarily focuses on the hypothalamic–pituitary–gonadal axis. However, until now, the direct effects of leptin on the testes during development from infancy to adulthood remained unclear. The aim of the present study was to explore the effects and molecular mechanisms that underlie leptin’s action in the testes during sexual maturation.

**Methods:**

We used a monosodium glutamate (MSG)-treated mouse model to assess the effects of endogenous hyperleptinemia on the development of the testes from infancy to adulthood. Then, a variety of reproductive parameters were measured, including the concentration of testosterone, the weight and volume of the testicles, the diameter of the seminiferous tubules, and numbers of spermatogonia, spermatocytes, sperm, Leydig cells and offspring. In addition, we assessed the direct role of leptin and suppressor of cytokine signalling 3 (SOCS3)/phosphorylated signal transducer and activator of transcription 3 (pSTAT3) on the testes in vitro.

**Results:**

Testosterone secretion exhibited a diverse response: a low concentration of leptin induced testosterone secretion, and a high concentration inhibited testosterone secretion both in vivo and in vitro. A variety of reproductive parameters decreased in hyperleptinemic mice, including the weight and volume of the testicles, the diameter of the seminiferous tubules, and the numbers of spermatocytes, sperm, Leydig cells and offspring. The amount of spermatogonia was also elevated. The development of the testes was partially recovered after hyperleptinemia withdrawal. A high concentration of leptin induced SOCS3 expression and inhibited pSTAT3 expression in the testes.

**Conclusions:**

The results indicated that MSG-induced hyperleptinemia directly affects testicular structure and function and that SOCS3/pSTAT3 played an important role in this process. These results also indicated the importance of monitoring and controlling leptin levels in obese male children. SOCS3 is a potential therapeutic target for leptin-induced dysgenesis.

## Background

Childhood obesity has become a worldwide problem, and its prevalence is increasing [1]. Studies of obese individuals with high levels of leptin suggest that hyperleptinemia is related to the body fat [2]. Many metabolic and hormonal processes are significantly influenced by changes in leptin secretion [3,4].

Leptin, which is the product of the obese (*ob*) gene, is produced primarily by adipose tissue and plays a pivotal role in the control of food intake and energy expenditure [5]. The effects of leptin are mediated by its specific receptor (LEPR) in target tissues. In the central nervous system, particularly in the basomedial hypothalamus, the leptin receptor mRNA has a site of high expression [6,7]. Since the discovery of leptin in 1994, most studies have described the role of leptin in the regulation of a series of endocrine systems and in obesity. Additionally, Donato et al. reviewed that humans and mice that lack leptin or the leptin receptor develop profound obesity and become infertile [8]. Giovambattista et al. reported that leptin plays an inhibitory role in reproductive functions [9]. Thus, the relation between leptin and reproduction is a cause for concern.

MSG treatment can increase the leptin level and change the testicular morphology [10]. Additionally, MSG treatment significantly diminishes plasma testosterone levels in the rat [11]*.* MSG treatment is considered to be an inducing hyperleptinemia model. LEPR is expressed in mouse testes, including Leydig cells, which produce testosterone [12,13]. Tena-Sempere et al. reported that leptin significantly decreased testosterone secretion in incubated testicular tissue from adult rats and that leptin inhibited luteinising hormone and follicle-stimulating hormone secretion in incubated pituitaries from adult male rats [14]. These findings suggest that the actions of leptin on the reproductive system are complex and probably occur at different levels of the hypothalamic–pituitary–gonadal axis. However, the effects of leptin on the testes during development from newborn to adult have not been clarified.

Suppressor of cytokine signalling 3 (SOCS3), which is an important member of the suppressors of cytokine signalling superfamily, has been identified as a mediator of central leptin resistance. Ob-Rb is the long form of the leptin receptor, has identical extracellular and transmembrane domains, and is crucial for leptin action. Leptin can induce SOCS3 via signal transducer and activator of transcription 3 (STAT3)-mediated transcription, after which SOCS3 inhibits Ob-Rb signalling [15,16]. In this mechanism, SOCS3 acts as a feedback inhibitor of the JAK–STAT pathway, which activates leptin signalling through the inhibition of the phosphorylation of STAT3 [17-19]. Therefore, SOCS3 is considered to play a role in the response to leptin treatment. It has been reported that SOCS3 negatively regulates leptin signalling and plays an important role in mediating leptin sensitivity in neurons [20]. However, whether SOCS3 is involved in the effects of leptin on the testes is unknown.

The effects of high leptin levels on the male reproductive system are unclear. The present study aimed to explore the effects and underlying molecular mechanisms of leptin action on the testes during mouse maturation. By MSG treatment, we observed the effects of increasing leptin levels on the development of testes of mice from infancy to adulthood. We analysed the effects on the testes by increasing leptin levels through periodic and persistent treatments. Using in vitro experiments with isolated testes, we excluded the involvement of the hypothalamus and the pituitary gland and confirmed the direct effects of leptin on the testes. We also used gene silencing and SOCS3 overexpression to examine whether the SOCS3–pSTAT3 signalling pathway, which is one of multitudinous potential pathways, plays a role in testosterone production in the testes.

## Methods

### Animals and treatments

Six-week-old BALB/c male and female mice were obtained from the Animal Centre of Sichuan University. Mice were bred and maintained under standard housing conditions in the Animal Facility of the West China Hospital, Sichuan University. Any experimental research that was reported in the manuscript was performed with the approval of the ethics committee of the West China Hospital, Sichuan University on May 9th 2012, following number 043 (See Additional file 1). Female and male mice were allowed to mate in cages. Pregnant female mice were placed in transparent individual cages. The day the litters were born was defined as d0. Male offspring received an ip injection of MSG (4 mg/kg body weight; Sigma, St. Louis, MO), which was dissolved in a 0.9% NaCl solution (NS), at 10 o’clock each day from the first postnatal day (d0). Half of the pups from each litter were given an ip injection of the same volume of NS once per day at the same time point as the control group. The pups were injected until d14 (prepuberty), d28 (puberty), or d56 (adult) and were called 14MSG, 28MSG, and 56MSG or 14NS, 28NS, and 56NS group, respectively. To examine the recovery from treatment, some mice were treated with MSG for 14 days and then injected with NS instead of MSG to d56; these mice were called the 14MSG-d56 group. Another group was treated similarly, except that NS was started on d28; these mice were called the 28MSG-d56 group. Each group included 10 animals. The mice were sacrificed under anaesthesia at 10 o’clock, then the body weight was measured, and the plasma was obtained for measurement of leptin and testosterone concentrations. The epididymis adipose mass and the testes were removed immediately to weigh, and the volume of each testis was measured. Additionally, the left testis was frozen in liquid nitrogen and the right testis was fixed in 4% paraformaldehyde. The testis dimensions were measured using a linear calliper, and the testis volume was calculated using the formula: volume (cm^3^) = 4πabc/3, where a, b, and c are the diameters in the x-, y-, and z-axes, respectively.

In addition, for procreation observation, with the same treatment as 14MSG-d56, 28MSG-d56 and 56MSG, each MSG-treated male mouse was mated with three normal female mice. Each group included 10 MSG-treated male mice. The pregnant female mice were placed in transparent individual cages. The numbers of pups in the litter at the first birth were recorded, and the total number of offspring was divided by the number of total mated female mice to acquire the mean offspring number.

### Hormone measurement

The leptin level of mouse plasma was measured using a commercial ELISA kit from Boster Biological Engineering Co., Ltd. (Wuhan, China) according to the manufacturer’s instructions. Testosterone levels in plasma and in the medium of the testicular static incubation were detected using a commercial EIA kit from R & D systems, Inc. (Minneapolis, MN) according to the manufacturer’s protocol. Hormone determinations were conducted in triplicate.

### Static incubation of testicular tissue in vitro

To detect the direct effects of leptin on the testes, testicular tissue was incubated in vitro using previously described methods [14,21,22], with some modifications. Briefly, testes were obtained from d14, d28, and d56 mice that corresponded to the age points that were studied in vivo. The animals were killed by decapitation. The testes were harvested immediately, decapsulated, and cut into two halves of approximately equal size. Hemi-testes were incubated in 2 ml of Dulbecco’s modified Eagle’s medium (DMEM; Gibco), 100 units/ml penicillin, and 100 mg/ml streptomycin (Sigma) in a Dubnoff shaker (60 cycles/min) at 37°C under an atmosphere of 5% CO_2_/95% O_2._ After preincubation for 1 h, the medium was replaced with fresh medium or with medium that contained 10–100 nM recombinant mouse leptin (R & D systems, Inc. Minneapolis, MN, USA). Each experiment was composed of six independent hemi-testicular samples that were obtained from six normal mice. After a 180-min incubation, the supernatants were harvested, and the testosterone concentration was measured. Hemi-testes were also stimulated with 10 or 100 nM leptin for 24 h, and the testes from the d56 group were frozen in liquid nitrogen for the determination of SOCS3 and STAT3 expression levels.

### Testicular immunohistochemistry

The right testis of mice in each group was obtained and, to maintain integrity were fixed in 4% paraformaldehyde for 48 hours and embedded in paraffin. Then, the testes were sliced into 5-μm sections at the 1/4 and 1/2 level of the testis along the long diameter. The sections were processed for the immunohistochemical (IHC) detection of Leydig cells (LC), using a specific polyclonal antibody, 3β-hydroxysteroid dehydrogenase (3β-HSD), which was purchased from Santa Cruz Biotechnology, Inc (California, USA). The testes were incubated overnight at 4°C with a goat-anti-mouse 3β-HSD (1:400 dilution) primary antibody. Subsequently, the tissues were incubated with a biotinylated donkey-anti-goat immunoglobulin (1:800 dilution, Zhongshanjinqiao, China) secondary antibody. Then, the slides were counterstained with haematoxylin. A goat isotype IgG (1:400 dilution, Zhongshanjinqiao, China) was designed to be the corresponding native control. The two level sections per testis were counted for the Leydig cell number determinations, and ten different regions for each section were chosen within random fields (400× magnification). Additionally, the results for each area were calculated and recorded as the number of positive cells that immunostained for 3β-HSD of LC per field. Using the same counting method, the quantity of integrated seminiferous tubules per field was counted at 100× magnification. The numbers of spermatogonia, spermatocytes and sperm were also counted in seminiferous tubules according to previously described characteristics [23] at 400× magnification.

### Overexpression and knockdown of SOCS3

Testes tissues were removed quickly in aseptic conditions, and equal sizes were completely masticated in complete DMEM medium with 10% foetal bovine serum. Then, the tissue was passed through a mesh to achieve comparable fragments. The tissue fragments (±1 mm^3^) were resuspended, and 70 mg of tissue fragments were plated into the wells of a six-well plate, which contained 2 ml of DMEM medium without antibiotics. The tissue cells were transfected with an expression construct for SOCS3 (pcDNA3.1-SOCS3) for the overexpression of SOCS3 or with siRNA-SOCS3 for the silencing of SOCS3. The pcDNA3.1-SOCS3 vector was constructed by ligating SOCS3 full-length cDNA into the pCDNA3.1 vector (Invitrogen, Carlsbad, CA). SOCS3 siRNA and a negative control siRNA were purchased from the GenePharma Company (Shanghai, China). The respective sequences for SOCS3 siRNA and the negative control siRNA were as follows: SOCS3 siRNA: 5′-GAC CCA GTC TGG GAC CAA G-3′; negative control siRNA: 5′-UUC UUC GAA CGU GUC ACG UTT-3′. Transfection was mediated using the TurboFect Transfection Reagent (Thermo Fisher Scientific) according to the manufacturer’s protocol with minor change and the transfection method established by our team [24]. The tissue cells were harvested at 24 hours after transfection to detect the expression of SOCS3 and STAT3/pSTAT3, and the testosterone level in medium was measured.

### Western blotting

Western blot analysis was performed to measure the expression of SOCS3, STAT3 and phosphorylated-STAT3 (pSTAT3). Whole-cell protein extracts from cells were prepared using lysis buffer for 30 min on ice. Protein concentrations were determined using an assay kit (Bio-Rad, Hercules, CA). Then, 50 μg of protein lysates were loaded, separated by denaturing sodium dodecyl sulphate-polyacrylamide gel electrophoresis and transferred to a polyvinylidene difluoride membrane (Millipore, Billerica, MA). Membranes were incubated in blocking buffer (Tris-buffered saline containing 5% skim milk) for 1 h at 37°C, followed by hybridisation with a rabbit-anti-SOCS3 antibody, a rabbit-anti-STAT3 antibody, and a rabbit-anti-pSTAT3 (tyr-705) antibody (1:1000 dilution, Cell Signaling Technology, USA) or a rabbit-anti-β-actin antibody (1:100 dilution, Lab Vision, Fremont, CA) at 4°C overnight. Then, membranes were hybridised with a horseradish peroxidase-conjugated rabbit immunoglobulin G (1:5000 dilution, Santa Cruz Biotechnology) secondary antibody for 1 hour at room temperature. Protein bands were detected by chemiluminescence using a western blotting luminol reagent (Santa Cruz Biotechnology). Films were scanned using a Gel Doc™ EZ Imager (Bio-Rad) and the protein level was semi-quantified using the Quantity One 1D image analysis software 4.4.0 (Bio-Rad).

### Statistical analysis

The data were expressed as the mean ± SD. To evaluate the significant differences between two groups, the means were compared using Student’s t-test. Multiple group comparisons were performed using a one-way analysis of variance followed by Student-Newman-Keuls test to conduct multiple comparisons. The results were considered significant for p-values <0.05. These analyses were performed using the SPSS 13.0 software (SPSS, Chicago, IL, USA).

## Results

### MSG treatment augmented the weight of body fat and induced hyperleptinemia mice in a time-dependent manner

**Figure 1:**
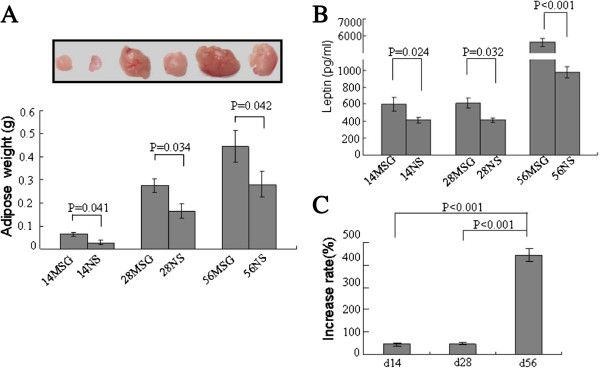
**MSG ip increased the weight of body fat and induced hyperleptinemia.** MSG or NS treatments were performed using intraperitoneal injections (ip) in mice from the first postnatal day (d0) to 14 days, who were called 14MSG or 14NS mice; to 28 days, who were called 28MSG or 28NS mice; or to 56 days, who were called 56MSG or 56NS mice. These selected times are representative of prepuberty, puberty and adulthood, respectively. The epididymal adipose was obtained from each MSG and NS-treated group **(A)**. The leptin levels in the plasma of MSG or NS- treated mice **(B)**. The rate of increased leptin level was assessed using MSG-treated mice compared with the corresponding NS-treated mice and dividing by the leptin concentration of NS-treated mice **(C)**.

To study the effects of hyperleptinemia and the time course of this effect in the mouse testes, male offspring were injected ip with MSG every day from d0 to d14 (prepuberty), d28 (puberty), or d56 (adult) (14MSG, 28MSG, and 56MSG groups, respectively). Although the body weight of MSG treatment mice was similar to that of NS treatment mice (Additional file 1: Figure S1), the weight of epididymal adipose in MSG treatment mice clearly increased (Figure 1A). The leptin level increased in MSG-treated mice compared with NS-treated mice (Figure 1B). However, the rate of increase for leptin concentrations in 14MSG and 28MSG group mice compared with 14NS and 28NS group mice was far lower than in the 56MSG group (Figure 1C). The results indicated that MSG could induced fat accumulation and obesity that produce hyperleptinemia.

### MSG treatment hindered the development of the testes and influenced the differential secretion of testosterone in vivo

**Figure 2:**
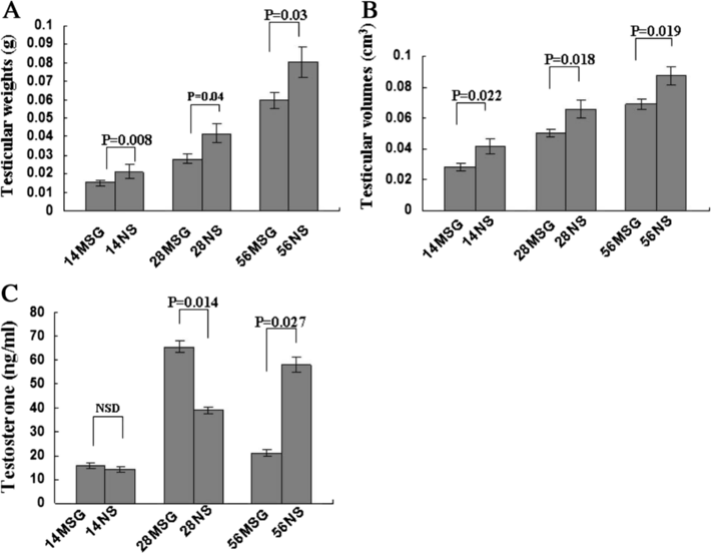
**MSG treatment reduced the weight and volume of the testes and differentially influenced testosterone secretion in vivo.** The pups were injected until d14 (prepuberty), d28 (puberty), or d56 (adult) with MSG or NS. Then the mice were sacrificed under anesthesia at 10 o’clock. The testicular weight and volume were measured for all MSG-treated and NS-treated mice **(A, B)** at all ages that were tested. ELISA analysis showed that the testosterone level of the 14MSG mouse plasma was slightly higher than that of the NS group; however, the difference was not significant (*p* > 0.05). Testosterone concentrations of the 28MSG mice increased compared with the 28NS-treated mice; however, the testosterone secretion in the 56MSG mice had a marked reduction (*p* < 0.05) **(C)**. NSD, non-significant difference.

**Figure 3:**
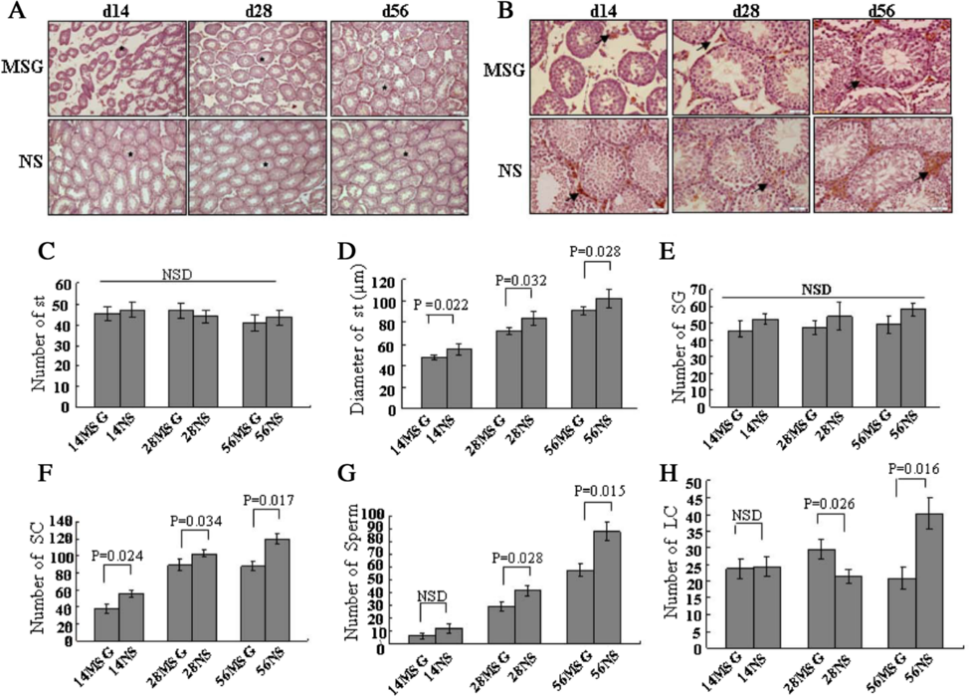
**MSG treatment hindered the development of the testes. A** and **B** are representative of the morphology of the seminiferous tubules (ST) of testes with IHC staining. Scale bar, 50 μm **(A)** and 3β-HSD positive Leydig cells with immunohistochemical staining. Scale bar, 20 μm **(B)**. The positive cells were stained brown. The positive cells were cytoplasmic positive and membranous positive. In the middle and bottom panels are shown the average number of ST **(C)**, the average diameter of ST **(D)** and the average number of 3β-HSD-positive Leydig cells per visual field **(H)**. The numbers of spermatogonia (SG), spermatocytes (SC), and sperm per seminiferous tubule were counted separately **(E, F, G)**. *Star,* seminiferous tubule; *Arrow,* 3β-HSD positive of Leydig cells; The means were compared using Student’s t-test. NSD, non-significant difference.

Testicular weight and volume are frequently used to evaluate the development of testes. In our results, testicular weight and volume decreased in all MSG-treated mice compared with NS-treated mice at all ages tested (Figure 2A, B). It is known that testosterone is the critical hormone for male development. As Figure 2C shows, the testosterone level was slightly, but not significantly (*p* > 0.05), higher in the 14MSG mice than in the 14NS mice. Interestingly, the testosterone concentration increased in the 28MSG mice compared with the 28NS mice (*p* < 0.05), but decreased markedly in the 56MSG mice (*p* < 0.05).As the Figure 3A and B shows, 3β-HSD IHC stained the Leydig cells in the testes. The number of seminiferous tubules (C) was not affected by MSG; however, the diameter of the tubules (D) was shortened and the arrangement was loosened in MSG-treated mice. The number of spermatogonia that were located adjacent to the basement membrane in the seminiferous tubule of MSG-treated mice compared with NS-treated mice had no significant difference (E). In addition, the quantity of spermatocytes at different stages of the seminiferous cycle and sperm in MSG-treated mice was lower than that in the NS group (F, G). However, the variable effects on testosterone concentration in vivo suggested that the MSG injection might affect testicular Leydig cells. The testicular Leydig cells were assessed by 3β-HSD IHC staining; 3β-HSD is a marker of Leydig cells, which secrete testosterone. MSG treatment decreased the number of Leydig cells in the d56 mice and increased the number of these cells in the d28 mice, but had no effect on the d14 mice (Figure 3H).

### Development of testes was recovered partly by decreasing leptin concentration by the withdrawal of MSG treatment

**Figure 4:**
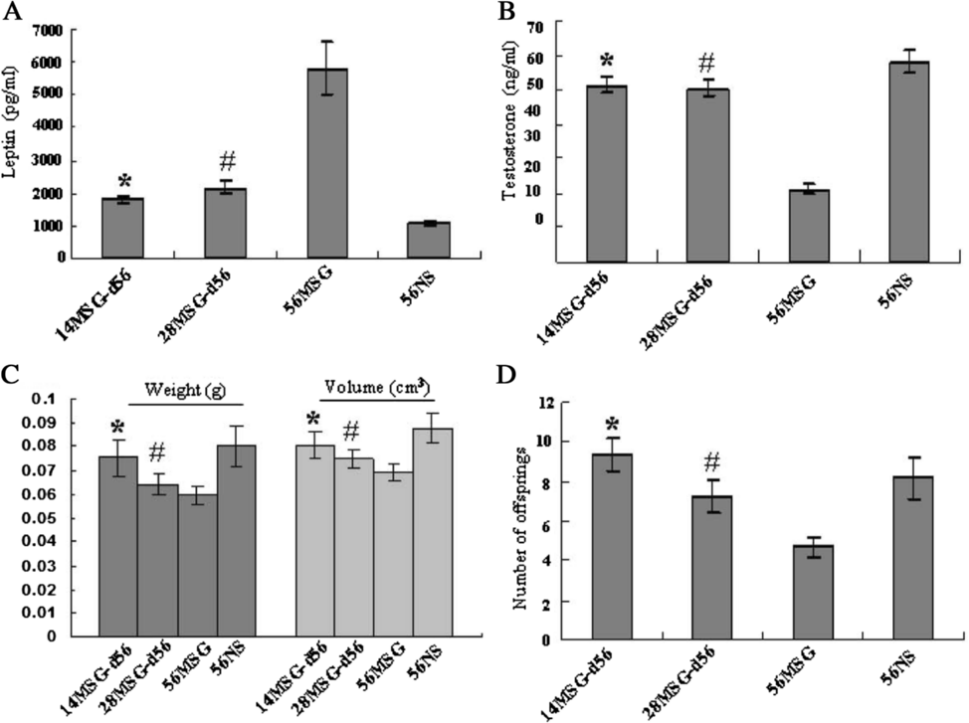
**Change in testosterone and offspring was recovered partly by decreasing leptin concentration by the withdrawal of MSG treatment.** Mice were treated with MSG from d0 to d14 or d28, and then injected with NS instead of MSG to d56; these groups are called 14MSG-d56 and 28MSG-d56, respectively. The leptin level of the mouse plasma was measured by ELISA **(A)**. Testosterone level of plasma was detected by EIA **(B)**. The methods to measure the testicular weight and volume **(C)** were described in the Materials and Methods. The total number of offspring was divided by the number of mated female mice to obtain the average number of offspring for each female mouse **(D)**. The means were compared using a one-way analysis of variance followed by Student-Newman-Keuls test to conduct multiple comparisons. *, p < 0.05 compared with the 56MSG-treated mice group; #, p < 0.05 compared with the 56NS-treated mice group; NSD, non-significant difference.

**Figure 5:**
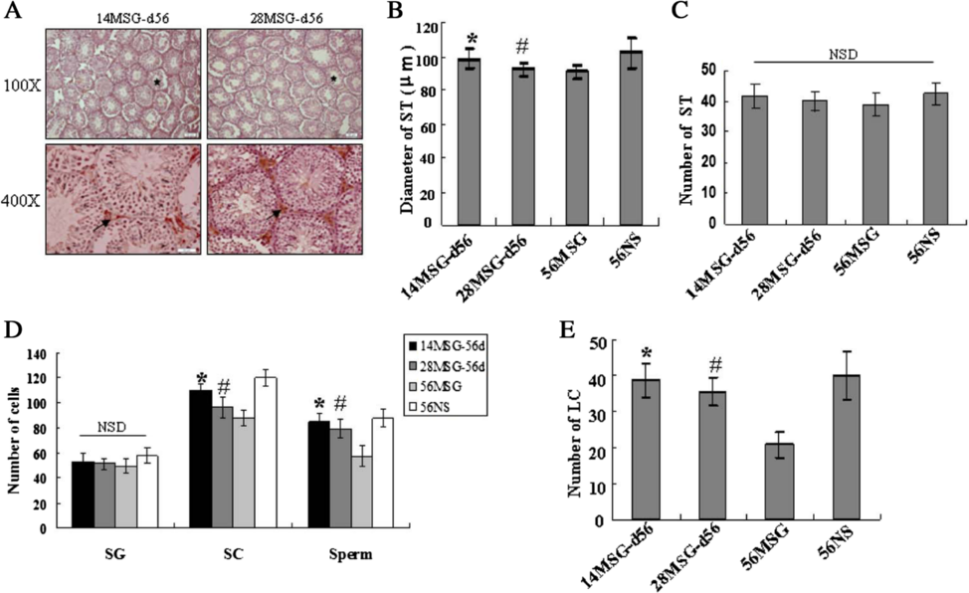
**Development of testes was recovered partly by decreasing leptin concentration by the withdrawal of MSG treatment.** In addition to the alterations in Figure 4, some other parameters were influenced by MSG treatment. Seminiferous tubules and Leydig cells **(A)**, diameter of seminiferous tubules **(B)**, number of seminiferous tubules **(C)**, number of spermatogonia (SG), spermatocytes (SC), sperm **(D)** and leydig cells **(E)** were analysed as described above. The means were compared using a one-way analysis of variance followed by Student-Newman-Keuls test to conduct multiple comparisons. *, p < 0.05 compared with the 56MSG-treated mice group; #, p < 0.05 compared with the 56NS-treated mice group; NSD, non-significant difference.

Mice were treated with MSG from d0 to d14 or d28, and then injected with NS instead of MSG to d56; these groups were called 14MSG-d56 and 28MSG-d56, respectively. As shown in Figure 4A, the plasma leptin level was decreased by stopping MSG treatment. The leptin concentrations remained higher in the 14MSG-d56 and 28MSG-d56 groups compared with the 56NS group, but were lower than in the MSG-d56 group. These results show that the leptin concentration recovered partially after the withdrawal of MSG treatment. Although the testosterone were lower in MSG-treated mice than in NS-treated mice for all d56 mice, the testosterone levels were enhanced in the 14MSG-d56 and 28MSG-d56 mice compared with the MSG-d56 mice (Figure 4B).Similar to the patterns of change in testosterone, the testicular weight, volume (Figure 4C) and the number of the offspring (Figure 4D) were recovered by the withdrawal of MSG, although these variables differed between the 14MSG-d56 and 28MSG-d56 groups compared with the 56MSG and 56NS groups. From the pictures of testes with 3β-HSD IHC staining (Figure 5A), other developmental parameters such as the diameter of seminiferous tubules (Figure 5B), and the number of spermatocytes, sperm and Leydig cells (Figure 5D, E), showed the same pattern of partial recovery as for the weight and volume of testes. However, the number of seminiferous tubules and spermatogonia (Figure 5C, D) did not change significantly after the withdrawal of MSG.

### Leptin directly influenced testosterone secretion by the testes in vitro, and MSG had no obvious effects

**Figure 6:**
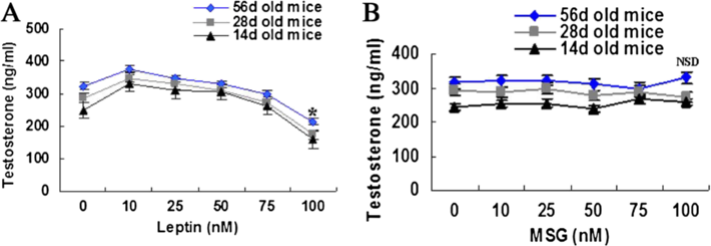
**Leptin directly influenced testosterone secretion by the testes in vitro, and MSG had no obvious effects.** Equal decapsulated testes were incubated with several concentrations of leptin, and the testosterone of the supernatant was detected by EIA. In low concentrations (<10 nM), leptin stimulated testosterone secretion, and high concentrations (>10 nM) of leptin inhibited this secretion, regardless of the age of the mice **(A)**. Testosterone secretion had no significant variations when the testes were stimulated with different concentrations of MSG in vitro **(B)**. Statistical method was performed by one-way analysis of variance. *, p < 0.05 compared with other concentrations; NSD, non-significant difference compared with other concentrations.

To analyse the direct effects of leptin on testosterone secretion, testicular tissues from mice of different ages were incubated and stimulated with different concentrations of leptin in vitro. Lower concentrations (10 nM) stimulated testosterone secretion, and higher concentrations (100 nM) inhibited this secretion, regardless of the age of the mice from which the testes were obtained (Figure 6A). This result may explain, at least in part, why the testosterone concentration increased in the MSG-d28 mice, which received a relatively low dosage of leptin, but the testosterone concentration decreased in the MSG-56d mice, which had received a higher dosage of leptin. In addition, different concentrations of MSG were used to stimulate testes; these concentrations were identical to those concentrations that were used in the leptin experiments. The results indicated that there was no significant difference in testosterone secretion among the MSG treatment groups (Figure 6B).

### High concentration of leptin induced the expression of SOCS3 and inhibited the phosphorylation of STAT3

**Figure 7:**
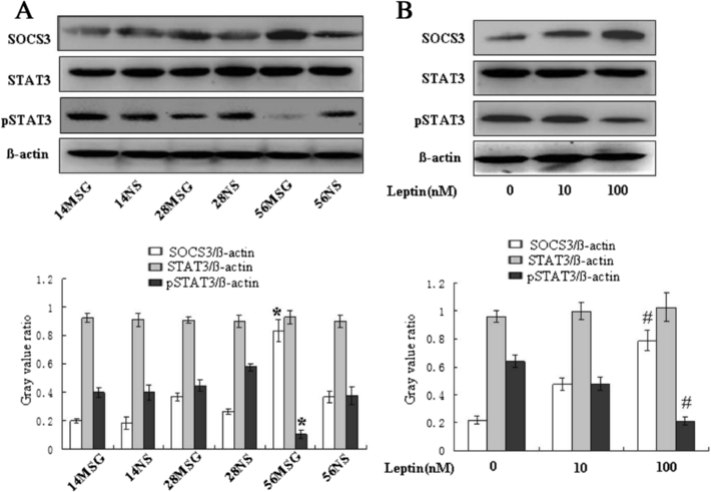
**High concentration of leptin induced the expression of SOCS3 and inhibited the phosphorylation of STAT3.** The expression of SOCS3 and STAT3/pSTAT3 were detected by western blotting. Representative expressions of SOCS3 and STAT3/pSTAT3 for testes of each age point were shown **(A)**. Expression of SOCS3 in d56 MSG-treated mice testes increased compared with 56NS mice, whereas SOCS3 expression had no obvious change in the d14 or d28 groups. However, the expression of pSTAT3 was opposite that of SOCS3. Total STAT3 had no obvious change. To further confirm the effects of leptin on the expression of SOCS3 and on the phosphorylation of STAT3, leptin concentrations of 0, 10 and 100 nM were used to stimulate the incubation of testes **(B)**. The lower panel represented graphs of western blotting band intensity. Statistical method was performed by a one-way analysis of variance followed by Student-Newman-Keuls test to conduct multiple comparisons. *, p < 0.05 compared with 56NS-treated mice group; #, p < 0.05 compared with 0 nM leptin-treated mice group.

SOCS3 is induced by leptin in the hypothalamus and is the main regulator of adrenocorticotropic hormone secretion [16]. SOCS3 also regulates the JAK–STAT pathway [17-19]. To explore the role of SOCS3 and STAT3/phosphorylation of STAT3 (pSTAT3) in the testicular leptin signalling pathway, we used western blot analysis to detect the expression of SOCS3 and STAT3/pSTAT3 in the testes. As shown in Figure 7A, the expression of SOCS3 and STAT3/pSTAT3 did not differ significantly between the MSG-d14 and MSG-d28 mice compared with their corresponding NS-treated groups. However, both increased expression of SOCS3 and decreased expression of phosphorylated-STAT3 (pSTAT3) were evident in the testes of the MSG-d56 mice. STAT3 had not changed (Figure 7A). These findings suggest that a high concentration of leptin affects the expression of SOCS3 and the phosphorylation of STAT3 in the testes.To further study the role of a high leptin concentration in the expression of SOCS3 and pSTAT3, we incubated testicular tissue with 0, 10 or 100 nM leptin respectively. The higher concentration of leptin induced the expression of SOCS3 and inhibited the level of pSTAT3, whereas the lower concentration of leptin had no effect, and STAT3 remained stable (Figure 7B). These findings confirmed that a high concentration of leptin affects SOCS3 expression and the pSTAT3 level.

### Testosterone secretion was influenced by SOCS3 expression and by the pSTAT3 level

**Figure 8:**
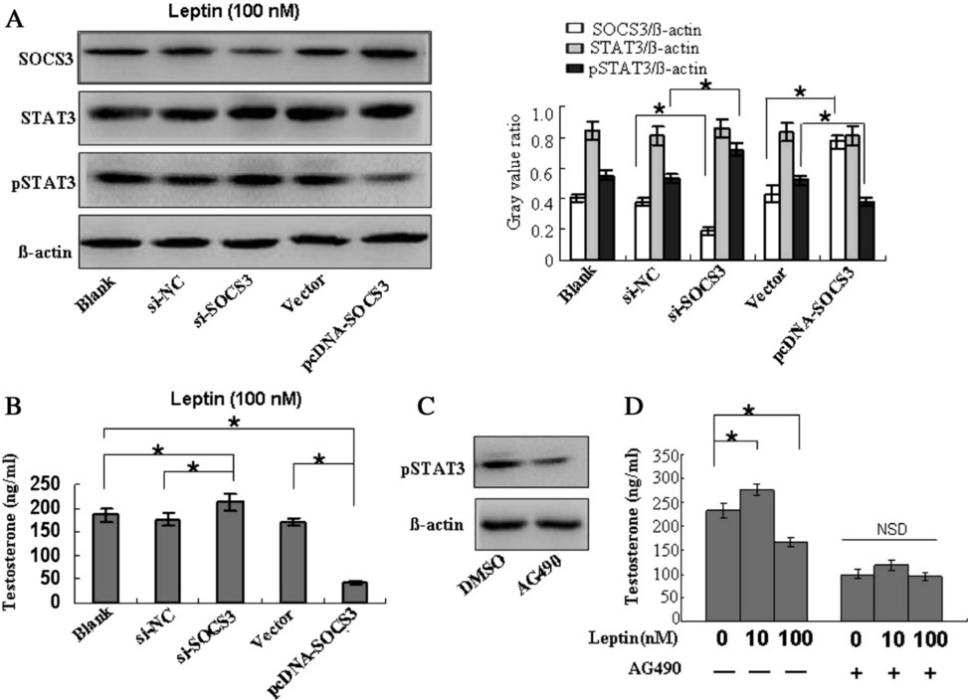
**Testosterone secretion was influenced by the expression of SOCS3 and pSTAT3 level. (A)** The expression of SOCS3 and STAT3/pSTAT3 after stimulation of 100 nM leptin was detected by western blotting. **(B)** The expression of SOCS3 after stimulation of 100 nM leptin could regulate the secretion of testosterone. **(C)** It was confirmed that AG490 (20 μM) could block STAT3 phosphorylation. **(D)** The induced role of low concentrations of leptin and inhibited role of high concentrations of leptin both dropped compared with the DMSO-alone group. The means were compared using a one-way analysis of variance followed by Student-Newman-Keuls test to conduct multiple comparisons. *, p < 0.05; NSD, non-significant difference. The photograph is representative of three independent experiments. Each column and bar represents the mean ± SD of three independent experiments.

Because a high concentration of leptin (i.e., 100 nM) affected both the secretion of testosterone and the expression of SOCS3, we used this concentration in further studies of the relation between testosterone secretion and SOCS3 expression. The downregulation of SOCS3 expression using si-SOCS3 increased STAT3 phosphorylation. The stimulation of testicular tissue with 100 nM leptin increased the testosterone level in the si-SOCS3 group compared with the blank and negative control (si-NC) groups. These findings suggest that the downregulation of SOCS3 expression weaken the inhibitory role of high concentrations of leptin. Conversely, the pSTAT3 level was reduced by the upregulation of SOCS3 expression (pcDNA-SOCS3), and testosterone secretion in cells that were stimulated with 100 nM leptin was lower in the pcDNA-SOCS3 group compared with the blank and vector groups (Figure 8A, B).Because the phosphorylation of STAT3 appears to play a role in SOCS3 function, we further studied the role of pSTAT3 in testosterone secretion in the testes. We used AG490 to block the phosphorylation of STAT3 in leptin-stimulated testicular tissue (Figure 8C). Testosterone levels with stimulation of 10 or 100 nM leptin in AG490-blocked groups were lower than the control groups (Figure 8D). The result indicated that the inducer role of low concentrations of leptin and the inhibitory role of high concentrations of leptin both decreased compared with the DMSO-alone group. These findings confirmed that SOCS3 and pSTAT3 play critical roles in testosterone secretion by the testes.

## Discussion

Although the negative effects of obesity on reproductive function were first documented many years ago by Hippocrates [25], the mechanism underlying this relation has not yet been thoroughly investigated. The relation between leptin and reproduction has received much attention in recent years. Some studies have reported that leptin primarily targets the hypothalamus [18,26] and that reproductive function is influenced by the hypothalamic–pituitary–gonadal axis [8,27,28]. Herrid et al. reviewed that leptin has direct effects on the regulation of testicular development and reported that testosterone production is diverse in the immature and adult mouse testes [29,30]. However, little is known regarding the potential mechanisms of the direct action of leptin on the testicular structure and function at different ages in mice. The observation that leptin receptors are present in male gonadal tissue [12,13] provided a basis for our study. Accordingly, we conducted this study to explore whether leptin regulates the testes directly during maturation from infancy to adulthood and, if so, to study the mechanism that is responsible for this effect.

Methods that are used to establish models of obesity in animals involve neuroendocrine, dietary, or genetic manipulation [31]. MSG treatment is one neuroendocrine method to induce obesity. Several studies had established and successfully utilised the MSG-induced obesity model [10,32,33], which results in a syndrome that is characterised by obesity and hypogonadism [34]. On the other hand, MSG is a polar solute whose dose in the blood vessels of tissue, including the hypothalamus and testes, is limited to <1%. Meanwhile, the blood/brain barrier (BBB) restricts and adjusts the flux of substrates between the circulation and the central nervous system. As MSG crosses lipid cellular membranes or is transported by selective BBB carriers, MSG is depleted [35]. In addition, our results exclude the direct role of MSG in testosterone production in vitro. Compared with dietary methods, ip MSG treatment is much easier to control and is more likely to induce obesity. To maintain the endogenic leptin state, we could exclude the methods of genetic change and direct leptin injection.

The mice were treated with MSG each day from d0 to d14, d28, or d56, which represent the prepubertal, pubertal, and postpubertal stages of maturation, respectively. Consistent with a previous report [9], we observed alterations that accompanied MSG-induced endogenous hyperleptinemia through the course of development; MSG treatment elevated leptin concentration on d14 and d28, and markedly elevated leptin concentration on d56. We could observe that epididymal adipose accumulated more in MSG-treated mice; however, there was no significant difference in the body weight, which might be limited by the short period of observation [36]. Although some studies revealed that the ip administration of MSG in animals showed a reduction in dietaty intake and body weight [9,11]. The common aspect of these studies was that the total fat mass significantly increased, which paralleled with concentrated leptin circulation in the blood [10,37]. Additionally expression of LEPR is detected in most tissues, including hypothalamus and testes. Circulatory leptin can cross the blood/brain or blood/testes barrier to combine with LEPR. Thus intra-testicular concentrations of leptin can mirror circulatory concentrations in part [38].

Testosterone, which is the major androgen, is produced by Leydig cells in the testes. To measure the effect of MSG on testicular function, we measured the plasma testosterone concentration at corresponding time points. We found that testosterone secretion in MSG-treated mice displayed an increasing trend from prepuberty to puberty (d14 to d28), but then decreased from puberty to adulthood (d28 to d56). Our results from the in vitro incubation of testicular tissue showed that testosterone secretion was stimulated by a lower concentration (10 nM) of leptin, but was inhibited by a higher concentration (100 nM) of leptin. This result is consistent with those results from our in vivo study of MSG-treated mice. These findings suggest that the production of testosterone is affected by the timing of MSG treatment. MSG-treatment to d28 slightly increased leptin and testosterone production, but that with continued MSG treatment to d56, the high level of leptin inhibited testosterone production. This result indicates that MSG-induced hyperleptinemia affected Leydig cells, which are the major cells that produce testosterone, through a direct or indirect approach.

The decreases in testicular weight and volume showed that hyperleptinemia impaired testicular development; however, this effect was reversible in part by the withdrawal of MSG. The testicular weight appears to be the most sensitive indicator of drug toxicity [39], and the testicular volume is regarded as an index of spermatogenesis [40-42]. Although MSG induced testosterone secretion during prepuberty and puberty, there were also negative effects on testis development, which were obvious through adulthood. The histopathological analysis of the testes showed that hyperleptinemia had negative effects on the testicular structure from infancy; persistent MSG treatment caused changes in the testicular structure, such as looser seminiferous tubule distribution and smaller tubule diameter. Additionally, MSG treatment prevented the proliferation and differentiation of spermatocytes and sperm. Variations in the seminiferous tubules have a negative effect on reproduction [11]. However, the testicular structure and function recovered partly after withdrawal of the MSG treatment, including increased testosterone level, testicular weight, volume, number of offspring, diameter of seminiferous tubules, number of spermatocytes, sperm and Leydig cells. To our knowledge, this report is the first to demonstrate such reversible changes. Leydig cell steroidogenesis is one functional parameter in reproduction [43,44]. The enzymatic complex 3β-HSD, which is in the endoplasmic reticulum and mitochondria, plays an essential role in the biosynthesis of testosterone and is detected in Leydig cells [45,46]. We found a reduced number of 3β-HSD-positive Leydig cells in the testes of mice that were treated with MSG continuously from infancy to adulthood (MSG-d56 group) and an increased number in the MSG-d28 group; however, these variations were not found in the MSG-d14 group. The number of offspring that were produced by normal female mice that mated with MSG-treated male was lower in MSG-treated than in NS-treated mice. These results show that MSG-treated hyperleptinemia has adverse effects on testicular development, function and fecundity.

In this study, it has been shown that the testicular expression of SOCS3 is regulated by leptin. SOCS3 was previously identified as a potential mediator of central leptin resistance. SOCS3 negatively regulates leptin signalling and plays important roles in mediating leptin sensitivity, glucose homeostasis, and energy expenditure [25]. Consistent with some studies, leptin induces the expression of SOCS3 and then SOCS3 negatively regulates the leptin level [18]. When leptin resistance emerged, the regulatory role was attenuated. Then, leptin concentration suddenly increased and induced the expression of SOCS3. To rule out the contribution of other regulatory signals, we used a static in vitro system to incubate testicular tissue, and we confirmed that leptin induced the expression of SOCS3 directly. In addition, some studies have reported that SOCS3 regulates the JAK–STAT pathway in the hypothalamus. Our results clearly showed that SOCS3 and pSTAT3 were also expressed in the testes. By down- and upregulating SOCS3, we confirmed that SOCS3 regulates testosterone secretion in the testes through the STAT pathway. This result has demonstrated that SOCS3 regulation and STAT3 phosphorylation play important roles in testosterone secretion and, consequently, affect the development of the testes.

## Conclusions

In conclusion, we provide evidence that MSG-induced hyperleptinemia affects the testicular structure and function at different maturation stages in mice in a concentration- and time-dependent manner. Our in vitro experiments also confirmed that leptin directly regulates testosterone secretion in the testes and excluded the contribution of other regulatory signals from the hypothalamic–pituitary–gonadal axis. Continuous MSG treatment of mice for 56 days caused leptin accumulation. The withdrawal of MSG treatment led to the partial recovery of testicular structure and function, which demonstrated that the effects of leptin could be at least partly reversed. Our findings suggest that the plasma leptin level should be estimated and controlled as early as possible in obese male children. We have also shown that SOCS3 plays a critical role in the leptin-induced inhibition of testosterone secretion in the testes. Our study has identified a regulatory mechanism for testosterone production and has suggested that SOCS3 is a potential therapeutic target for treating leptin-induced dysgenesis.

## Abbreviations

3β-HSD: 3β-hydroxysteroid dehydrogenase; ip: Intraperitoneal; IHC: Immunohistochemical; LC: Leydig cells; MSG: Monosodium glutamate; NS: 0.9% NaCl solution; pSTAT3: Phosphorylated signal transducer and activator of transcription 3; SOCS3: Suppressor of cytokine signalling 3; STAT3: Signal transducer and activator of transcription 3; ST: Seminiferous tubules.

## Competing interests

The authors declare that they have no competing interests.

## Authors’ contributions

All authors participated in the design, interpretation of the studies, analysis of the data and review of the manuscript; MY, GZH, PL and LGH conducted the experiments; MY and JL wrote the manuscript; JZ, FL, KL and BG performed the hormone assay; and LZ and WS performed data analyses. All authors read and approved the final manuscript.

## Supplementary Material

Additional file 1: Figure S1 The body weight of mice had no obivious alterations by MSG treatment. Male offspring were injected ip with MSG or NS every day from d0 to d14 (prepuberty), d28 (puberty), or d56 (adult) (14MSG, 28MSG, 56MSG and 14NS, 28NS, 56NS groups, respectively). The body weight of MSG treatment mice compared with NS group was no significant difference. Statistical method was performed by Student’s t-test. NSD, non-significant difference. Click here for file
