# Supplementary figures and images for: Hyperleptinemia directly affects testicular maturation at different sexual stages in mice, and suppressor of cytokine signaling 3 is involved in this process
Source: Reprod Biol Endocrinol. 2014 Feb 6;12:15. doi: 10.1186/1477-7827-12-15 (PMC4015707; doi:10.1186/1477-7827-12-15)

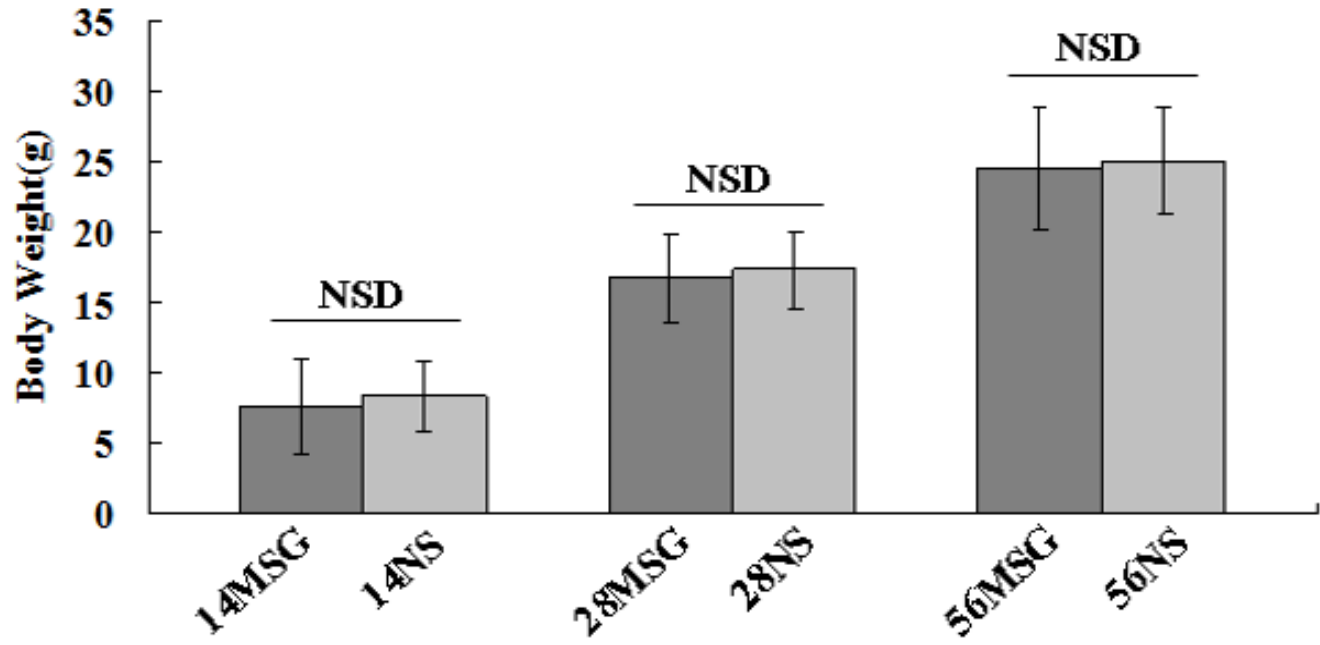

Supplement: Additional file 1: Figure S1 — The body weight of mice had no obivious alterations by MSG treatment. Male offspring were injected ip with MSG or NS every day from d0 to d14 (prepuberty), d28 (puberty), or d56 (adult) (14MSG, 28MSG, 56MSG and 14NS, 28NS, 56NS groups, respectively). The body weight of MSG treatment mice compared with NS group was no significant difference. Statistical method was performed by Student’s t-test. NSD, non-significant difference. [file 1477-7827-12-15-S1.pdf]
